# Designing a Palliative Care Pathway for Enhanced Dementia Care

**DOI:** 10.1093/geroni/igaf122.2221

**Published:** 2025-12-31

**Authors:** Christine Jensen, Jenny Inker

**Affiliations:** Riverside Martha W. Goodson Center, Williamsburg, Virginia, United States; Virginia Commonwealth University, Richmond, Virginia, United States

## Abstract

Riverside Health has developed a new palliative care pathway to serve as an innovative standard of person-centered care for persons living with dementia (PLWD) and their care partners. Building on an existing interdisciplinary palliative care team (PCT) of a physician, nurse practitioner, and intake/nurse navigator, the positions of social worker, geriatric pharmacist, and spiritual care advisor were added to the team. The PCT have completed extensive training in dementia-specific palliative care, including risks for those who live alone, and those with developmental disabilities. Further, educational programs, in the form of microlearning lessons, have been designed and delivered to PLWD, their care partners, the PCT, and community-based partners. These microlearning lessons (brief bursts of targeted educational content) highlight the key elements of palliative care, which are often inaccurately equated with hospice care, and ways to address stigma associated with both a dementia diagnosis and enrolling in palliative care. The enhanced PCT has served more than 175 PLWD and their family caregivers, with resulting increases in quality of life and well-being for both PLWD and care partners. PLWD and care partners have expressed gratitude for social work support in connecting to resources, as well as the meaning that is provided through engaging with a spiritual care advisor. Additionally, comprehensive medication reviews provided by the geriatric pharmacist have resulted in a decrease in medication burden and an increase in adherence. Application of palliative care principles and lessons learned will be referenced for other health systems to consider.

